# Clustering Molecules at a Large Scale: Integrating Spectral Geometry with Deep Learning

**DOI:** 10.3390/molecules29163902

**Published:** 2024-08-17

**Authors:** Ömer Akgüller, Mehmet Ali Balcı, Gabriela Cioca

**Affiliations:** 1 Faculty of Science, Department of Mathematics, Mugla Sitki Kocman University, Muğla 48000, Turkey; oakguller@mu.edu.tr; 2 Faculty of Medicine, Preclinical Department, Lucian Blaga University of Sibiu, 550024 Sibiu, Romania; gabriela.cioca@ulbsibiu.ro

**Keywords:** deep learning, spectral geometry, molecular topology, clustering algorithms

## Abstract

This study conducts an in-depth analysis of clustering small molecules using spectral geometry and deep learning techniques. We applied a spectral geometric approach to convert molecular structures into triangulated meshes and used the Laplace–Beltrami operator to derive significant geometric features. By examining the eigenvectors of these operators, we captured the intrinsic geometric properties of the molecules, aiding their classification and clustering. The research utilized four deep learning methods: Deep Belief Network, Convolutional Autoencoder, Variational Autoencoder, and Adversarial Autoencoder, each paired with k-means clustering at different cluster sizes. Clustering quality was evaluated using the Calinski–Harabasz and Davies–Bouldin indices, Silhouette Score, and standard deviation. Nonparametric tests were used to assess the impact of topological descriptors on clustering outcomes. Our results show that the DBN + k-means combination is the most effective, particularly at lower cluster counts, demonstrating significant sensitivity to structural variations. This study highlights the potential of integrating spectral geometry with deep learning for precise and efficient molecular clustering.

## 1. Introduction

A computational technique called molecular clustering groups molecules according to their structural or functional similarities. This process entails analyzing various molecular properties, such as their geometric, topological, or chemical attributes, in order to identify clusters of molecules that share common features [1,2,3,4]. By grouping similar molecules together, researchers can uncover patterns and relationships that might not be immediately apparent when examining individual molecules.

In the fields of chemistry and biology, molecular clustering plays an important role in numerous applications. In the field of chemoinformatics, molecular clustering has been extensively used for chemical diversity in the compound acquisition, analysis of high-throughput screening (HTS) results for lead discovery, and virtual screening. These applications have broadened to include biological information and improved validation techniques [5]. Molecular clustering is important for understanding the underlying biological mechanisms, such as in the study of hemagglutinin (HA) molecules in influenza type A. Using advanced clustering methods, researchers can estimate challenging critical parameters associated with molecular clusters, which are important for cell biology and biophysics [6]. Clustering techniques are also used to analyze molecular dynamics simulation trajectories, grouping protein conformations based on geometric and kinetic similarities. This aids in understanding protein interactions and structural dynamics [7]. In computational biology, clustering algorithms help in the classification of gene expression patterns and the identification of disease pathways. Ensemble clustering techniques improve robustness and reliability in analyzing high-dimensional biological data [8].

Clustering large molecular datasets presents several significant challenges due to molecular data’s inherent complexity and high dimensionality. One of the primary challenges is the sheer volume of data. Modern molecular databases can contain millions of compounds, each represented by a vast array of features, including geometric, topological, and chemical descriptors. Processing such large datasets requires substantial computational resources and efficient algorithms capable of handling high-dimensional spaces [9,10,11,12].

The diversity of molecular structures is another challenge. Molecules can vary widely in size, shape, and composition, leading to a highly heterogeneous dataset. This diversity makes it difficult to identify meaningful clusters, as the similarity measures used for clustering must be robust enough to handle a wide range of molecular features [13,14,15]. Additionally, the presence of noise and outliers in the data can further complicate the clustering process. Noise can arise from experimental errors or inaccuracies in molecular representations, while outliers are molecules that do not fit well into any cluster, potentially skewing the clustering results. The selection of appropriate features for clustering is also a challenge. Molecular data often include a large number of features, many of which may be redundant or irrelevant for clustering purposes. Identifying the most informative features that accurately capture the similarities and differences between molecules is essential for effective clustering. Moreover, different clustering algorithms may perform variably depending on the feature set used, adding another layer of complexity to the task.

Traditional clustering methods, such as k-means, hierarchical clustering, and DBSCAN, have several limitations when applied to complex molecular structures. One significant limitation is their reliance on predefined distance metrics, such as Euclidean distance, which may not adequately capture the intricate similarities and differences between molecular structures. The chosen distance metric may inaccurately group molecules with similar geometric or chemical properties [16]. k-means clustering, for instance, assumes that the data points within each cluster are evenly distributed around a central point in a spherical shape. However, this assumption often does not hold true for molecular data, where the clusters can have irregular shapes and distributions. For large and diverse datasets, determining the number of clusters in advance can be challenging with this method [17]. Hierarchical clustering, while providing a detailed dendrogram of nested clusters, is computationally intensive and may become impractical for enormous datasets due to its high time complexity [18].

Density-based methods, such as DBSCAN, can find clusters of any shape and deal with noise well. However, they need the right parameters (like epsilon and minimum points), which can be hard to get right for molecular datasets that are very complicated [19,20,21]. Additionally, DBSCAN may struggle with varying density clusters, which are common in molecular data. Traditional clustering methods also often fail to leverage the rich structural information available in molecular datasets. They typically do not account for the complex relationships between atoms and bonds within molecules, which can be significant for accurate clustering. This limitation underscores the need for advanced techniques that can integrate structural, geometric, and topological information to improve clustering performance.

Spectral geometry is a field of mathematics that studies the properties of geometric structures using the spectrum of differential operators, such as the Laplace–Beltrami operator. In the context of molecular analysis, spectral geometry provides powerful tools for understanding the intricate shapes and surfaces of molecules [22,23,24]. By examining the eigenvalues and eigenvectors of these operators, researchers can capture geometric features that are invariant to transformations like rotation and translation. For comparing and analyzing molecular structures, which may have different orientations in space but possess the same intrinsic geometry, this invariance is significant.

Deep learning has emerged as a powerful tool for clustering large and complex datasets, offering significant advancements over traditional methods. By leveraging neural networks, deep learning methods can automatically learn and extract meaningful features from raw data, which are often high-dimensional and intricate. This capability is particularly valuable in clustering, where the goal is to group similar data points without predefined labels. One of deep learning’s primary roles in clustering is to transform data into a lower-dimensional latent space, where clustering becomes more tractable. Neural networks, especially deep ones, can capture complex, nonlinear relationships within the data, making them ideal for clustering applications. This transformation helps to uncover the underlying structure of the data, facilitating more accurate and meaningful cluster formation. Several studies have demonstrated the efficacy of Deep Belief Networks (DBNs) in molecular clustering. For instance, Ref. [25] showed that DBNs could effectively model the distribution of high-dimensional data, making them suitable for clustering tasks. Researchers have used DBNs in molecular studies to cluster molecules based on their structural features, revealing meaningful patterns and relationships [26,27,28,29].

We have employed Convolutional Autoencoders (CAEs) in the context of molecular clustering to extract features from molecular surfaces. Studies have shown that CAEs outperform traditional feature-extraction methods by preserving spatial hierarchies and capturing intricate details of molecular surfaces [30,31,32,33]. Studies have shown that CAEs outperform traditional feature-extraction methods by preserving spatial hierarchies and capturing intricate details of molecular surfaces. This results in clusters that better reflect the underlying structural similarities and differences.

Research has shown that Variational Autoencoders (VAEs) are highly effective for molecular clustering. For instance, Ref. [34] introduced VAEs and demonstrated their potential for unsupervised learning tasks. Molecular analysis uses VAEs to cluster molecules by learning latent representations that capture the underlying structure of the data. Researchers have found that VAEs can create smooth latent spaces that make clustering work better [35,36,37]. This lets us find new molecular groups and learn more about molecular diversity.

We have employed Adversarial Autoencoders (AAEs) in molecular clustering to enhance the separation and compactness of clusters. For example, Ref. [38] demonstrated that AAEs could learn more structured latent spaces compared to traditional autoencoders. Subsequent molecular analysis studies applied AAEs to cluster molecules based on their structural and functional attributes. These studies have found that AAEs can generate well-separated clusters that align closely with known molecular classifications, highlighting their potential to improve clustering accuracy and reveal new molecular insights [39,40].

The spectral geometric methodology employed in this study involves several key steps to transforming molecular structures into a form suitable for deep learning-based clustering. First, we extract each molecule’s solvent-accessible surface (SAS), which represents the boundary that the solvent molecule would perceive. This surface is then discretized into a triangulated mesh using algorithms such as Delaunay triangulation or Marching Cubes to create a network of triangles that accurately represent the 3D shape of the molecule.

Next, we apply the Laplace–Beltrami operator to these triangulated meshes. The Laplace–Beltrami operator is a differential operator that generalizes the Laplacian notion to curved surfaces. By solving the eigenvalue problem for this operator, we obtain a set of eigenvalues and eigenvectors that capture the intrinsic geometric properties of the molecular surfaces. The eigenvectors provide a spectral decomposition of the surface, encapsulating essential geometric features that are invariant to transformations like rotation and translation.

To manage the computational complexity and ensure the relevance of the features, we perform weighted sampling based on the discrete mean curvature at each vertex of the triangulated mesh. This approach prioritizes vertices with significant curvature, capturing the most structurally relevant features of the molecular surfaces.

We chose spectral geometry for feature extraction because it uniquely captures the intrinsic geometric and topological properties of molecular surfaces, which are crucial for accurate and meaningful clustering. Unlike traditional methods that rely on predefined distance metrics, such as Euclidean distance, spectral geometry utilizes the eigenvalues and eigenvectors of the Laplace–Beltrami operator to provide a comprehensive, invariant representation of molecular shape. This approach allows us to move beyond simple geometric descriptors and harness a more detailed and nuanced depiction of molecular structures. The inherent invariance of spectral geometry to transformations such as rotation and scaling ensures that the extracted features are robust, consistent, and suitable for reliable comparisons across diverse molecular datasets. By focusing on the fundamental geometric characteristics, this method addresses the limitations of traditional clustering techniques, which often fail to account for the complex relationships between atoms and bonds within molecules.

Integrating spectral geometry with deep learning methods leverages the complementary strengths of both approaches, resulting in a more powerful and accurate clustering methodology. Spectral geometry provides a rich, detailed representation of molecular surfaces, capturing both global and local geometric features. At the same time, deep learning excels at modeling complex, nonlinear relationships within high-dimensional data. This integration allows us to effectively utilize the intricate geometric properties of molecules as input for advanced deep learning models, which can uncover deeper patterns and relationships within the data that traditional methods may overlook. This combined approach enhances clustering accuracy, improves the handling of high-dimensional and complex molecular data, and enables the automatic extraction of meaningful features without manual intervention. As a result, it holds the potential to discover novel molecular groups and offers deeper insights into molecular diversity and structure–function relationships.

However, this integration also presents challenges, including the computational complexity associated with both spectral geometry and deep learning models. These challenges require substantial computational resources and the development of efficient algorithms to manage the demands of the process. Additionally, the integration process itself must be carefully managed, with meticulous tuning and optimization to ensure that the spectral features are effectively utilized by the deep learning models. Despite these challenges, this study demonstrates how the integration of spectral geometry with deep learning successfully addresses the limitations of traditional clustering methods, particularly in handling high-dimensional molecular data and providing robust, accurate feature-extraction techniques. This approach not only improves clustering accuracy but also enhances our understanding of molecular structures and their functional implications.

## 2. Methodology

### 2.1. Spectral Geometry of Molecular Surfaces

The van der Waals surface is the outermost boundary of a molecule. It is determined by the van der Waals radii of the atoms that make up the molecule. Every atom provides its own distinct sphere, which is described by its empirical radius. These spheres collectively determine the border. This surface is essential for comprehending molecular interactions, including steric hindrance, molecular recognition, and docking processes in the field of drug discovery [41,42,43,44]. The molecule’s physical boundaries are clearly defined, creating a spatial structure that allows other molecules to bind at specific sites. This binding can occur by direct contact or through induced fit mechanisms. The van der Waals surface is an important concept in computational chemistry for understanding molecular interactions. It is typically defined as the surface where the electron density drops to 0.001 electrons/Å^3^, while not a primary input for simulating molecular dynamics or predicting stability, the van der Waals surface helps in visualizing and analyzing molecular shapes and interactions. These simulations often require detailed representations of molecular geometries to evaluate how molecules approach and interact with each other, especially in biological systems. Understanding the van der Waals surface can assist chemists and biologists in anticipating the energy dynamics of molecular collisions and reactions, thereby enhancing our knowledge of the fundamental principles governing biochemical pathways.

The van der Waals surface of a molecule is represented by the outer boundary formed by the collection of spherical atoms. The van der Waals surface $S_{VdW}$ of a molecule can be expressed as the collective union of spheres, denoted as

(1) $$VDW=\overset{n}{\underset{i=1}{⋃}}\{x\in R^{3}:∥x-c_{i}∥\leq r_{i}\},$$

where $c_{i}$ and $r_{i}$ are the center and van der Waals radius of the *i*-th atom, respectively.

While the van der Waals surface provides a valuable depiction of a molecule’s shape based on atomic radii, it does not account for the molecule’s interaction with its solvent environment. To address this, the concept of the solvent-accessible surface (SAS) is introduced. The SAS takes into consideration a solvent molecule, typically modeled as a sphere, which ‘probes’ the surface of the molecule. This approach is crucial for understanding how molecules interact with their environment, particularly in aqueous solutions where solvent accessibility plays a significant role in molecular behavior and stability.

The SAS is defined through the use of a probe sphere that simulates the solvent molecule. This sphere rolls over the surface of the molecule, defined by the van der Waals radii of its constituent atoms. The SAS is thus the locus of the center of the probe sphere as it maintains contact with the van der Waals surface without penetrating any atom’s van der Waals radius. Mathematically, the SAS can be defined as follows

(2) $$SAS=\overset{n}{\underset{i=1}{⋃}}\{p\in R^{3}:\min_{i}∥p-c_{i}∥=r_{i}+r\}.$$

Here, *r* is the radius of the probe sphere, representing the solvent molecule. The equation ensures that the shortest distance from any point *p* on the SAS to the center $c_{i}$ of any atom in the molecule is equal to the sum of the atom’s van der Waals radius $r_{i}$ and the probe sphere is radius *r*. This effectively models the boundary beyond which the solvent can approach the molecule without penetrating its atomic radii, thus providing a more realistic representation of molecular interaction sites in a solvent environment.

SAS is an important tool in molecular modeling that provides information on the interaction between molecules and their surrounding solvent. Conventionally, these surfaces are regarded as smooth manifolds, which, although mathematically elegant, frequently necessitate intricate analytical methods for thorough examination [45,46]. In order to enhance computational analysis and take advantage of advanced computational tools such as deep learning, it is extremely advantageous to shift from continuous to discrete representations.

Within the field of computational geometry, discrete differential geometry offers a structured approach to estimating the characteristics of smooth surfaces using discrete elements like triangulations. By modeling the SAS as a mesh composed of triangles, we transform the continuous geometry issue into a problem that involves combinations and permutations. The mathematical representation of this transformation is denoted as follows:

(3) $$SAS\to SAS_{triangulated}=\left(V,E,F\right),$$

where *V* represents the vertices of the mesh, *E* represents the edges, and *F* represents the faces generated by these edges. Each vertex in this representation serves as an approximation of a point on the original smooth SAS, while the faces collectively serve as an approximation of the surface’s topology and geometry. We use the Computational Geometry Algorithms Library (CGAL) program to calculate the values of vertices, edges, and faces specified in Equation (3), ensuring accurate and reliable mesh processing for our analysis.

The Laplace–Beltrami operator is a fundamental tool in discrete differential geometry, extending the classical Laplacian to curved surfaces. This operator is particularly valuable for analyzing scalar fields defined on these surfaces, such as those related to the shape functions of molecular structures. In our study, we focus on these shape-related scalar fields to gain insights into the geometric properties of molecular surfaces. Unlike other potential scalar fields, such as temperature or electrostatic potential, which may vary based on external conditions or interactions, the shape functions are intrinsic to the molecular geometry itself. By applying the Laplace–Beltrami operator to these scalar fields, we can directly assess the fundamental geometric characteristics of the molecules, which is critical for understanding molecular interactions and stability in biophysical and pharmaceutical contexts. This localized analysis, centered on shape functions, is essential for investigating how molecular surfaces interact with their environment, particularly in complex biochemical processes.

In our study, the discrete version of the Laplace–Beltrami operator is applied to the triangulated solvent-accessible surfaces ($SAS_{triangulated}$) of molecules. The operator is discretized to operate on the vertices of the triangulated mesh, allowing us to perform localized geometric analysis. The discrete Laplace–Beltrami operator is defined as

(4) $$\Delta_{LB}f\left(v\right)=\frac{1}{A(v)}\sum_{(u,v)\in E}w_{uv}\left(f(u)-f(v)\right),$$

where f(v) represents a scalar function defined on the vertices of the mesh, A(v) denotes the area associated with vertex *v* (computed as one-third of the sum of the areas of the triangles adjacent to *v*), and $w_{uv}$ are the edge weights, which are determined using the cotangent of the angles opposite the edge (u,v) in the two adjacent triangles, as follows:

(5) $$w_{uv}=\frac{1}{2}\left(\cot\alpha_{uv}+\cot\beta_{uv}\right),$$

where $\alpha_{uv}$ and $\beta_{uv}$ are the angles opposite the edge (u,v) in the adjacent triangles. These cotangent weights provide a robust approximation of the curvature and ensure that the operator faithfully represents the underlying geometry of the molecular surface.

The matrix element ${\left(\Delta_{LB}\right)}_{ij}$ between vertices *i* and *j* is given by

(6) $${\left(\Delta_{LB}\right)}_{ij}=\left\{\begin{array}{cc} -\frac{w_{ij}}{A_{i}}, & \mathrm{if}\;i\;\mathrm{and}\;j\;\mathrm{are}\;\mathrm{adjacent}\\ \sum_{k\neq i}\frac{w_{ik}}{A_{i}}, & \mathrm{if}\;i=j\\ 0, & \mathrm{otherwise} \end{array}\right..$$

Once the Laplace–Beltrami operator is constructed, we perform spectral decomposition to analyze the molecular surface. This is performed by solving the eigenvalue problem, as follows:

(7) $$\Delta_{LB}\phi_{k}=\lambda_{k}\phi_{k},\;\;\mathrm{for}\;k=1,2,\ldots ,N,$$

where $\lambda_{k}$ are the eigenvalues and $\phi_{k}$ are the corresponding eigenvectors. The eigenvalues $\lambda_{k}$ provide information about the geometric properties of the surface, such as its global shape and localized features. The eigenvectors $\phi_{k}$ represent the modes of surface deformation and are key to capturing the detailed geometric structure of the molecular surface.

In our methodology, the eigenvectors $\phi_{k}$ obtained from the Laplace–Beltrami operator are combined into a matrix Φ, where each column of Φ corresponds to an eigenvector, as follows:

$$\Phi =[\phi_{1}\;|\;\phi_{2}\;|\;\cdots \;|\;\phi_{N}],$$

where *N* is the number of selected eigenvectors. This matrix Φ serves as the input for our deep learning methods. The combined matrix effectively encodes the geometric information of the molecular surface, allowing the deep learning models to learn from both the global shape and local features represented by the eigenvectors.

The deep learning models then utilize this matrix Φ to perform clustering tasks by feeding the matrix into a neural network. This matrix, which encapsulates the eigenvectors of the Laplace–Beltrami operator, serves as a rich, high-dimensional representation of the molecular surfaces. Through this process, the neural networks can learn to identify complex patterns, relationships, and underlying structures within the data that traditional clustering methods often miss. The deep learning models are particularly adept at capturing nonlinear interactions and hierarchical features, which are crucial for accurately grouping molecules based on subtle geometric variations. By integrating spectral geometry with deep learning, we achieve a robust and detailed representation of molecular surfaces that leverages the strengths of both techniques, leading to more precise and meaningful clustering outcomes.

**Figure 1:**
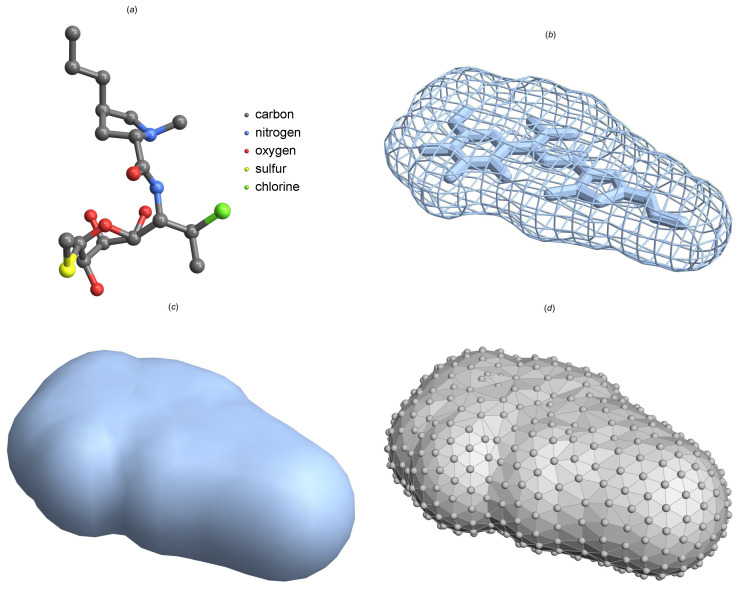
(**a**) A 3D plot of *clindamycin* molecule, (**b**) triangulated VDW for *clindamycin*, (**c**) SAS, and (**d**) $SAS_{triangulated}$ where the small spheres denote vertices.

Figure 1 displays the SAS and $SAS_{triangulated}$ geometries derived from the *clindamycin* molecule, which is also part of the dataset we will utilize. When creating SAS, a probe sphere is typically chosen to have a radius of approximately r≈1.4Å, which is similar in size to a water molecule. The mesh $SAS_{triangulated}$ consists of 857 vertices, 2311 edges, and 1466 faces.

**Figure 2:**
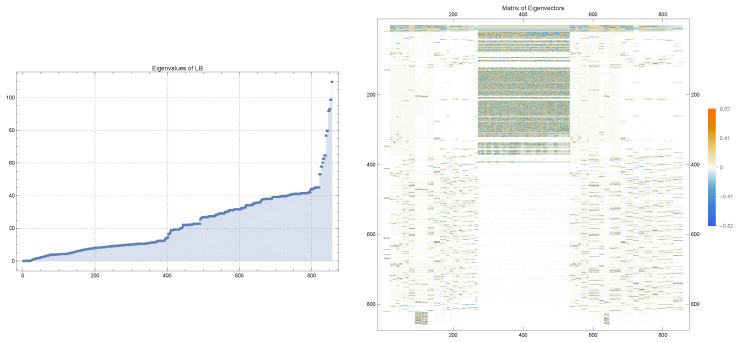
Eigenvalues (on the left) and the matrix of eigenvectors (on the right) resulting from the spectral decomposition of the Laplace–Beltrami operator.

Figure 2 displays the eigenvalues and the matrix of associated eigenvectors resulting from the spectral decomposition of the Laplace–Beltrami operator on $SAS_{triangulated}$.

**Figure 3:**
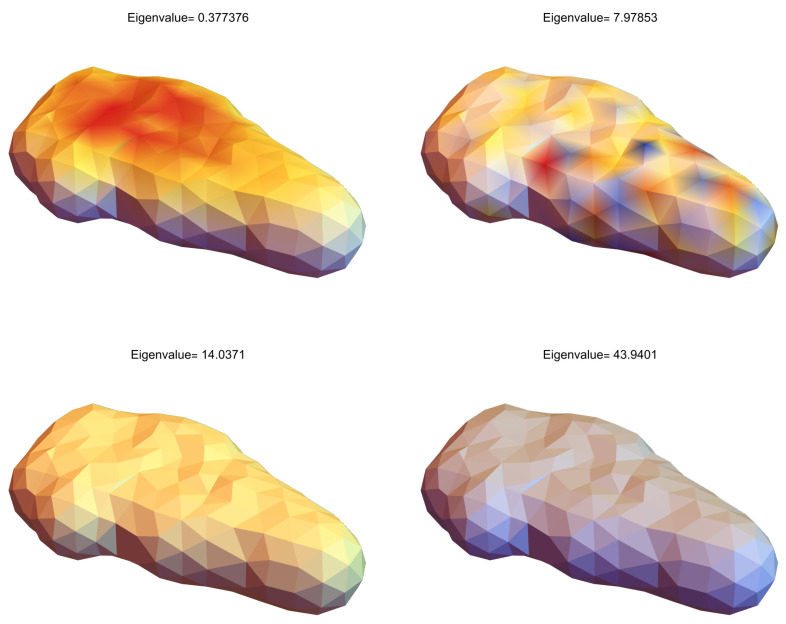
Eigenvectors with corresponding eigenvalues on $SAS_{triangulated}$.

Figure 3 displays the eigenvectors on the mesh region for visualization purposes. In this illustration, the color function is selected to be a temperature map that represents lower values with blue and higher values with red.

In this study, we aim to efficiently cluster a large-scale molecule library, consisting of approximately 50,000 molecules, screened against *Mycobacterium tuberculosis* mutant strains. Our methodology involves a multi-step process, leveraging both spectral geometry and deep learning insights to classify and cluster these molecules. A critical step in this process is the weighted sampling of vertices on the triangulated meshes obtained from the SAS surfaces of the molecules. To perform weighted sampling, we compute the discrete mean curvature at each vertex of the triangulated mesh. The discrete mean curvature H(v) at a vertex *v* is given by

(8) $$H\left(v\right)=\frac{1}{2}\sum_{u\in N(v)}\left(\cot\left(\alpha_{uv}\right)+\cot\left(\beta_{uv}\right)\right)∥vs.-u∥,$$

where N(v) denotes the set of vertices adjacent to *v*, and $\alpha_{uv}$ and $\beta_{uv}$ are the angles opposite the edge (v,u) in the two triangles sharing this edge. This formula leverages the cotangent weights, which provide a robust approximation of the curvature.

Given the discrete mean curvature values at each vertex, we proceed with the weighted sampling as follows:

- For each vertex v∈V, compute the discrete mean curvature H(v).
- Normalize the curvature values to obtain a probability distribution. Let $H_{\mathrm{norm}}\left(v\right)$ be the normalized curvature for vertex *v* with
  (9) $$H_{\mathrm{norm}}\left(v\right)=\frac{H(v)}{\sum_{u\in V}H\left(u\right)}.$$
- Using the normalized curvature values $H_{\mathrm{norm}}\left(v\right)$ as weights, we perform weighted sampling to select 200 vertices. This can be performed using a probabilistic approach where vertices with higher curvature values have a higher probability of being selected.

The probability P(v) of selecting a vertex *v* is given by

(10) $$P\left(v\right)=\frac{H_{\mathrm{norm}}\left(v\right)}{\sum_{u\in V}H_{\mathrm{norm}}\left(u\right)}.$$

We then use a random sampling algorithm, such as the weighted reservoir sampling, to select 200 vertices based on these probabilities.

The choice of 200 vertices was made to balance the computational efficiency of our methodology with the need to capture the most structurally significant features of the molecular surfaces. By focusing on a subset of vertices with the highest curvature values, we ensure that the key geometric characteristics of the molecules are well represented in our analysis. At the same time, limiting the number of sampled vertices to 200 allows us to perform the clustering and subsequent analysis in a computationally inexpensive manner, which is crucial given the large scale of our dataset. This approach ensures that our method remains scalable and practical, even when applied to extensive molecular libraries, without sacrificing the accuracy and relevance of the clustering results.

By utilizing discrete mean curvature as a weighting factor, the algorithm is able to effectively handle noise and small changes in the mesh geometry. This results in a dependable identification of important vertices. Discrete mean curvature characterizes the inherent geometric characteristics of the mesh by examining the local shape and surface fluctuations at every vertex. This metric is intrinsically less responsive to modest variations in the vertex positions that may result from numerical inaccuracies or slight deformations in the surface [47,48,49]. Therefore, by giving attention to vertices with elevated mean curvature values, we give priority to portions of the mesh that display significant geometric characteristics, such as edges, vertices, and face areas with high curvature. This method not only improves the stability of the sampling process but also guarantees that the chosen vertices accurately represent the most important structural features of the molecules. By maintaining these essential characteristics, we can attain a more precise and significant categorization, finally resulting in more dependable clustering outcomes within the framework of spectral geometry and deep learning research.

### 2.2. Clustering with Deep Learning

Within the scope of this work, deep learning clustering offers a potent and novel method for classifying huge molecular datasets. Even if they work well, traditional clustering techniques frequently have trouble handling the great dimensionality and complexity of molecular data, especially when attempting to handle the complicated geometric characteristics of molecule surfaces. Deep learning provides a major edge in this field because of its capacity to automatically learn and extract relevant characteristics from unprocessed data. We may convert the input data into a lower-dimensional representation that captures the fundamental structures and patterns seen in the molecules by utilizing neural networks.

The eigenvectors of the Laplace–Beltrami operator and the discretized triangulated meshes, which capture the geometric subtleties of every molecule, are the inputs of learning for the deep learning models used in this work. By identifying spontaneous groupings within the dataset without the need for predetermined labels, our unsupervised learning method enables us to obtain insights into the structural similarities and differences between the molecules. The operational concepts of these methods can be mathematically delineated as follows.

Let $X=\{x_{1},x_{2},\ldots ,x_{n}\}$ denote the dataset consisting of *n* high-dimensional data points. Each data point $x_{i}$ belongs to $R^{d}$, which represents a *d*-dimensional space. The goal is to acquire knowledge of a lower-dimensional representation $Z=\{z_{1},z_{2},\ldots ,z_{n}\}$, where $z_{i}$ belongs to the set of real numbers $R^{k}$ and *k* is significantly smaller than *d*. This is accomplished using a sophisticated neural network, typically an autoencoder or a convolutional neural network, which can be defined by the encoding function $f_{\theta }$ that is determined by the parameter θ as

(11) $$z_{i}=f_{\theta }\left(x_{i}\right).$$

The network is trained to minimize a reconstruction loss $L_{\mathrm{recon}}$, ensuring that the lower-dimensional representations *Z* retain the essential information of the original data

(12) $$L_{\mathrm{recon}}=\frac{1}{n}\sum_{i=1}^{n}{∥x_{i}-g_{\phi }\left(f_{\theta }\left(x_{i}\right)\right)∥}^{2},$$

where $g_{\phi }$ is the decoding function parameterized by ϕ.

Once the feature extraction is complete, the lower-dimensional representations *Z* are used as input for clustering. The clustering algorithm, such as k-means or Gaussian Mixture Models (GMMs), aims to partition *Z* into *K* clusters. For k-means clustering, this involves minimizing the within-cluster sum of squares (WCSS)

(13) $$L_{k\text{-}\mathrm{means}}=\sum_{j=1}^{K}\sum_{z_{i}\in C_{j}}{∥z_{i}-\mu_{j}∥}^{2},$$

where $C_{j}$ denotes the *j*-th cluster and $\mu_{j}$ is the centroid of cluster $C_{j}$.

Deep learning-based clustering commonly uses a joint optimization technique to optimize both the feature extraction and clustering processes simultaneously [50,51,52,53]. The process is accomplished by merging the reconstruction loss and the clustering aim into a unified loss function

(14) $$L=L_{\mathrm{recon}}+\lambda L_{\mathrm{cluster}},$$

where $L_{\mathrm{cluster}}$ represents the clustering loss (e.g., k-means loss) and λ is a regularization parameter that balances the two components.

The optimization process occurs in a series of iterations, during which the neural network parameters θ and ϕ, as well as the clustering parameters (such as centroids $\mu_{j}$), are adjusted in order to minimize the joint loss function. This repeated process improves both the feature representations and the quality of the clusters at the same time. The optimization can be formulated as

(15) $$\theta^{*},\phi^{*},\mu^{*}=\arg\min_{\theta ,\phi ,\mu }L,$$

where $\theta^{*}$, $\phi^{*}$, and $\mu^{*}$ denote the optimized parameters.

By following these principles, deep learning-based clustering methods effectively capture complex patterns and structures in high-dimensional data, leading to more accurate and meaningful clustering outcomes. This approach is particularly advantageous in the context of our study, where the intricate geometric properties of molecular surfaces are crucial for understanding and categorizing the molecular dataset. This study employs five distinct deep learning classification methods that are based on either autoencoders or convolutional neural networks.

Deep Belief Networks (DBNs) are a kind of generative deep learning model that consists of numerous layers of Restricted Boltzmann Machines (RBMs). A Restricted Boltzmann Machine (RBM) is a neural network consisting of two layers, a visible layer *v* and a hidden layer *h*. The visible layer is directly coupled to the hidden layer. The DBN trains each layer of RBMs sequentially in a greedy, unsupervised manner.

The mathematical formulation of a single RBM is defined by the energy function

(16) $$E\left(v,h\right)=-\sum_{i}v_{i}a_{i}-\sum_{j}h_{j}b_{j}-\sum_{i,j}v_{i}W_{ij}h_{j},$$

where $v_{i}$ and $h_{j}$ are the visible and hidden units, $a_{i}$ and $b_{j}$ are their biases, and $W_{ij}$ are the weights between them. The probability of the network being in a particular state is given by

(17) $$P\left(v,h\right)=\frac{e^{-E(v,h)}}{Z},$$

where *Z* is the partition function. DBN training entails optimizing weights to maximize the likelihood of the training data. After undergoing training, the DBN can be utilized to convert the input data into a feature space with reduced dimensions, which is well suited for clustering purposes.

Convolutional Autoencoders (CAEs) are a specific kind of autoencoder that employs convolutional layers for the purpose of encoding and decoding data. They are highly efficient for jobs that involve image data, as they maintain the spatial structure of the input.

The CAE consists of an encoder $f_{\theta }$ and a decoder $g_{\phi }$. The encoder maps the input *x* to a lower-dimensional latent representation *z* as in Equation (11). The decoder reconstructs the input from *z* with

(18) $$\hat{x}=g_{\phi }\left(z\right).$$

The network is trained to minimize the reconstruction loss

(19) $$L_{\mathrm{recon}}=∥x-\hat{x}{∥}^{2}={∥x-g_{\phi }\left(f_{\theta }\left(x\right)\right)∥}^{2}.$$

Once trained, the encoder $f_{\theta }$ extracts meaningful features from the input data, which can then be clustered using traditional clustering algorithms.

Variational Autoencoders (VAEs) are a class of generative models that enhance the conventional autoencoder by including a probabilistic framework in the latent space. The encoder maps the input *x* to a distribution over the latent space

(20) $$q_{\phi }\left(z|x\right).$$

The decoder generates the reconstruction from samples drawn from this distribution

(21) $$p_{\theta }\left(x|z\right).$$

The VAE is trained to maximize the evidence lower bound (ELBO), which balances the reconstruction accuracy and the divergence between the approximate posterior $q_{\phi }\left(z|x\right)$ and the prior p(z)

(22) $$L_{\mathrm{VAE}}=E_{q_{\phi }\left(z|x\right)}\left[\log p_{\theta }\left(x|z\right)\right]-\mathrm{KL}\left(q_{\phi }\left(z|x\right)∥p\left(z\right)\right).$$

The learned latent representations *z* can be used for clustering.

Adversarial Autoencoders (AAEs) integrate the concepts of autoencoders and adversarial training to enforce a certain distribution on the latent space. The AAE consists of an encoder $f_{\theta }$, a decoder $g_{\phi }$, and a discriminator $D_{\psi }$. The encoder maps the input *x* to a latent representation $z=f_{\theta }\left(x\right)$. The decoder reconstructs the input from *z* as in Equation (18). The autoencoder is trained to minimize the reconstruction loss, again as in Equation (19).

Simultaneously, the discriminator $D_{\psi }$ is trained to distinguish between the true prior distribution p(z) and the distribution of encoded data $q_{\phi }\left(z|x\right)$. The adversarial loss for the discriminator is

(23) $$L_{D}=-E_{p(z)}\left[\log D_{\psi }\left(z\right)\right]-E_{q_{\phi }\left(z|x\right)}\left[\log\left(1-D_{\psi }\left(z\right)\right)\right].$$

The encoder is trained to fool the discriminator

(24) $$L_{\mathrm{enc}}=-E_{q_{\phi }\left(z|x\right)}\left[\log D_{\psi }\left(z\right)\right].$$

Through the process of optimizing these losses, the AAE guarantees that the latent space adheres to a specific distribution, making it appropriate for clustering.

Assessing the caliber of clustering outcomes is essential for verifying the efficiency of clustering algorithms and guaranteeing that the recognized clusters possess significance and utility. Clustering measurements offer numerical metrics to evaluate many characteristics of the clusters, including their compactness, separation, and general structure. These metrics facilitate the comparison of various clustering approaches, adjustment of algorithm parameters, and selection of the most suitable clustering solution for a given dataset. This subsection provides the mathematical definitions of many often used clustering metrics: Calinski–Harabasz index, Davies–Bouldin index, Dunn index, R-Squared, Silhouette Score, and Standard Deviation.

The Calinski–Harabasz index, alternatively referred to as the Variance Ratio Criterion, assesses the proportion of between-cluster dispersion to within-cluster dispersion. For a dataset *X* with *n* data points divided into *k* clusters, the index is defined as

(25) $$\mathrm{CH}=\frac{\mathrm{trace}(B_{k})}{\mathrm{trace}(W_{k})}\cdot \frac{n-k}{k-1},$$

where $\mathrm{trace}(B_{k})$ is the trace of the between-cluster dispersion matrix and $\mathrm{trace}(W_{k})$ is the trace of the within-cluster dispersion matrix. Greater values suggest more distinct clusters.

The Davies–Bouldin index quantifies the average similarity ratio between each cluster and its most similar cluster. For *k* clusters, the index is given by

(26) $$\mathrm{DB}=\frac{1}{k}\sum_{i=1}^{k}\max_{j\neq i}\left(\frac{s_{i}+s_{j}}{d_{ij}}\right),$$

where $s_{i}$ is the average distance between points in cluster *i* and the centroid of cluster *i*, and $d_{ij}$ is the distance between the centroids of clusters *i* and *j*. Smaller numbers suggest more effective grouping.

The purpose of the Dunn index is to find clusters that exhibit both compactness and well-separatedness. It is defined as

(27) $$\mathrm{Dunn}=\frac{\min_{1\leq i<j\leq k}d\left(C_{i},C_{j}\right)}{\max_{1\leq l\leq k}\delta \left(C_{l}\right)},$$

where $d(C_{i},C_{j})$ is the distance between clusters $C_{i}$ and $C_{j}$, and $\delta (C_{l})$ is the diameter of cluster $C_{l}$. Higher values indicate better clustering.

R-Squared quantifies the percentage of variation that is accounted for by the grouping. It is defined as

(28) $$R^{2}=1-\frac{W_{\mathrm{total}}}{T_{\mathrm{total}}},$$

where $W_{\mathrm{total}}$ is the total within-cluster sum of squares and $T_{\mathrm{total}}$ is the total sum of squares. Higher values indicate better clustering.

The Silhouette Score measures the degree of similarity between a data point and its own cluster in comparison to other clusters. For each data point *i*, the Silhouette Score is given by

(29) $$s\left(i\right)=\frac{b(i)-a(i)}{\max(a(i),b(i))},$$

where a(i) is the average distance between *i* and all other points in the same cluster, and b(i) is the minimum average distance between *i* and points in a different cluster. The overall Silhouette Score is the average of s(i) overall points. The numbers in the range of −1 to 1 represent the quality of clustering, with larger values indicating superior clustering.

In the context of clustering, the standard deviation can refer to the spread of points within each cluster. For a cluster *C* with *n* points, the standard deviation σ is given by

(30) $$\sigma =\sqrt{\frac{1}{n}\sum_{i=1}^{n}{\left(x_{i}-\mu \right)}^{2}},$$

where $x_{i}$ is a point in the cluster and μ is the mean of the cluster. Lower values indicate more compact clusters.

### 2.3. Testing the Topological Descriptors

In the context of clustering molecular data, understanding the influence of topological indices on the clustering results is essential for elucidating the structural and functional relationships among molecules. Topological indices provide a quantitative measure of the molecular graph’s topology, capturing various aspects of the molecular structure. To assess the impact of these topological indices on the clustering outcomes, we employ nonparametric statistical tests, specifically the Kruskal–Wallis, Conover, and Gehan tests. These tests are particularly suitable for our study as they do not assume a specific distribution of the data, making them robust for analyzing complex and potentially non-normal data distributions.

The Balaban J index is a topological index that reflects the branching of the molecular graph. It is defined as

(31) $$J=\frac{m}{n-1+\sum_{i=1}^{n}\sqrt{d_{i}}},$$

where *m* is the number of edges, *n* is the number of vertices, and $d_{i}$ is the degree of vertex *i*. The Balaban J index quantifies the total level of branching and complexity shown by the molecule.

The Schultz Molecular Topological Index (MTI) is a measure of molecular branching and connectivity. It is calculated as

(32) $$\mathrm{MTI}=\sum_{i=1}^{n}\left(d_{i}+1\right)\left(d_{i}-1\right),$$

where $d_{i}$ is the degree of vertex *i*. The MTI evaluates both the magnitude and the level of interconnection of each vertex in the molecular graph.

The Szeged index is a topological descriptor that accounts for the distances between pairs of vertices in a molecular graph. It is given by

(33) $$Sz=\sum_{e=(u,v)}n_{u}\left(e\right)\cdot n_{v}\left(e\right),$$

where *e* is an edge in the graph, $n_{u}\left(e\right)$ is the number of vertices closer to *u* than to *v*, and $n_{v}\left(e\right)$ is the number of vertices closer to *v* than to *u*.

The Xu index is another topological index that reflects the connectivity and structure of the molecular graph. It is calculated as

(34) $$Xu=\sum_{i=1}^{n}\left(d_{i}^{2}-d_{i}\right),$$

where $d_{i}$ is the degree of vertex *i*. The Xu index emphasizes the impact of vertex degrees on the overall molecular structure.

The molecular complexity index, based on Böttcher’s definition, captures the intricacy of the molecular structure. It is defined as

(35) $$\mathrm{BC}=\sum_{i=1}^{n}\left(V_{i}b_{i}d_{i}e_{i}s_{i}\right),$$

where $V_{i}$ is the number of valence electrons, $b_{i}$ is the total number of bonds to any atom with $V_{i}b_{i}>1$, $d_{i}$ is the number of chemically nonequivalent bonds to atoms with $V_{i}b_{i}>1$, $e_{i}$ is the number of different atoms in atom *i*’s microenvironment, and $s_{i}$ is the number of possible isomeric structures at position *i*. This index provides a comprehensive measure of the molecule’s structural complexity.

Molecular Graph Autocorrelation (MGA) is a descriptor used in cheminformatics to quantify the structural characteristics of a molecule by representing it as a graph. In this representation, atoms are depicted as vertices and bonds as edges. To compute MGA, a specific property is assigned to each vertex, such as atomic weight, electronegativity, or any other atomic characteristic relevant to this study. The autocorrelation function is then defined for a given distance *k* (path length) within the graph. This function typically sums the products of properties of vertex pairs that are separated by the distance *k* in the molecular graph. Mathematically, the autocorrelation function AC(k) can be expressed as

(36) $$AC\left(k\right)=\sum_{i,j}p\left(v_{i}\right)\cdot p\left(v_{j}\right),$$

where $v_{i}$ and $v_{j}$ are vertices with a graph distance *k* between them, and p(v) denotes the property assigned to vertex *v*.

To enhance the descriptor, the Shannon entropy of the autocorrelation function can be included. Shannon entropy provides a measure of the uncertainty or randomness associated with the distribution of the autocorrelation values. It is calculated as

(37) $$H=-\sum_{i}p_{i}\log\left(p_{i}\right)$$

where $p_{i}$ represents the normalized autocorrelation values for each distance *k*. This entropy value captures the complexity and variability of the molecular structure, adding another layer of information to the MGA descriptor. By incorporating Shannon entropy, the descriptor not only reflects how molecular properties are distributed across the graph but also how varied and complex these distributions are, offering deeper insights into the molecule’s structural and electronic features.

By utilizing nonparametric statistical tests, we can systematically evaluate the influence of topological indices on the clustering outcomes, offering valuable insights into the structural factors that determine molecular clustering. These tests enable us to compare the distributions of topological indices among several clusters without making any assumptions about the underlying data distribution, ensuring that our analysis is resilient and reliable. The Kruskal–Wallis test is used to assess if there are statistically significant variations in the median values of topological indices among the clusters, providing an overview of how these indices differ across the entire dataset.

The Conover post hoc test is employed to determine the specific clusters that exhibit significant differences from each other, allowing for a comprehensive analysis of the pairwise variations. This test is crucial for pinpointing which clusters have distinct structural characteristics, facilitating a deeper understanding of the factors driving the separation between clusters. The Gehan test further enhances our analysis by comparing survival distributions, enabling us to assess the longevity and persistence of specific structural characteristics within clusters. This adds another dimension to our understanding by highlighting how long particular features are maintained within the cluster structure.

These statistical tools collectively allow us to discover intricate connections between molecular structure and clustering patterns, elucidating the role of various topological properties in the formation and stability of clusters. This comprehensive approach not only confirms the clustering results but also emphasizes the importance of topological indices in comprehending molecular structure and its implications for clustering. By revealing these relationships, we can better understand the molecular determinants that influence clustering behavior.

In conclusion, these observations can guide the development of more efficient clustering algorithms and improve our understanding of the intricate details within extensive molecular datasets. By leveraging nonparametric statistical tests, we ensure a robust analysis that can adapt to the complex nature of molecular data.

## 3. Results and Discussions

### 3.1. Dataset

The Ref. [54] dataset, accessible through Chemical Genomic Software, encompasses comprehensive structure and function annotations for an extensive collection of 47,217 compounds. These compounds are meticulously encoded using the Simplified Molecular-Input Line-Entry System (SMILES), a notation that translates complex molecular structures into concise ASCII strings. This encoding not only captures the connectivity and stereochemistry of molecules but also facilitates computational analysis by providing a standardized format for molecular representation. Each compound within the dataset is richly annotated with detailed atomic and bond information, encompassing attributes such as atomic numbers, formal charges, bond types, and lengths. We used this approach to cluster approximately 50,000 chemicals previously selected for a large-scale chemical-genetic screen against the bacterium *Mycobacterium tuberculosis*. Chemical-genetic interaction profiles (CGIPs) were created using *M. tuberculosis* mutant strains (hypomorphs), providing a detailed landscape of potential antibacterial activity. This work offers an in-depth analysis of a large-scale chemical library successfully utilized to identify compounds with potential antibacterial properties, highlighting the dataset’s application in discovering novel therapeutic agents.

**Figure 4:**
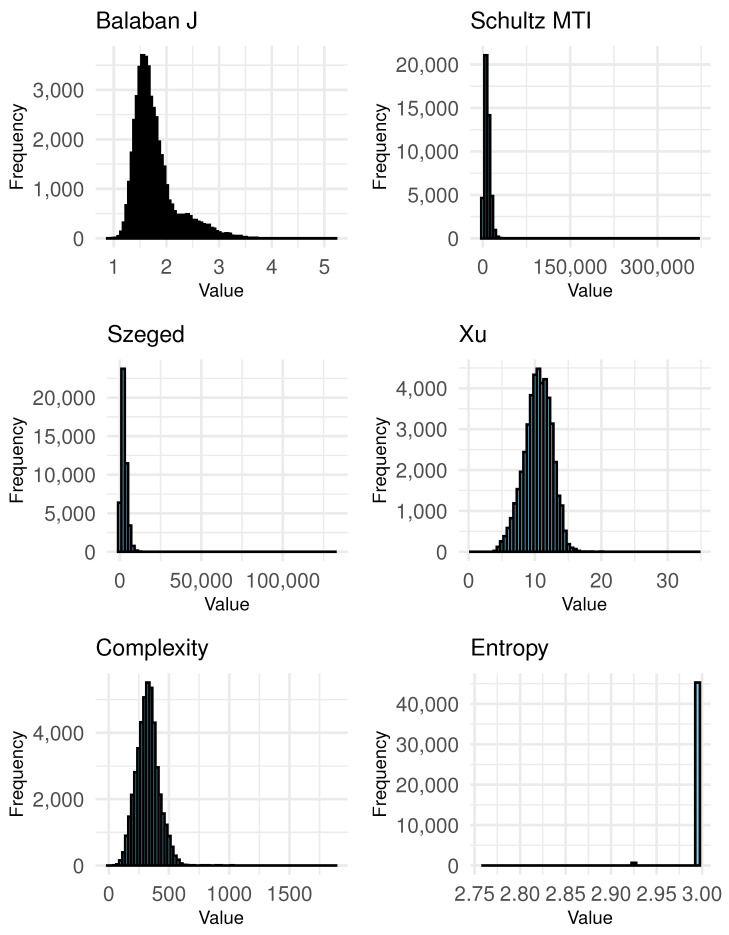
Distributions of the topological descriptors.

Additionally, we use six topological molecular descriptors for each compound. These descriptors are crucial for a myriad of applications, including chemical analysis, machine learning, and drug discovery. The dataset’s versatility allows for the exploration of chemical diversity and distribution, aiding researchers in understanding the structural and functional landscapes of these compounds. By leveraging graph representations and advanced feature engineering techniques, this dataset serves as a pivotal resource for both cheminformatics and computational biology, enabling sophisticated modeling and predictive analyses. In Section Figure 4, we depict the distributions of topological descriptors that are calculated for this study.

In Figure 4, the distribution of the Balaban J index is right-skewed, with most molecules having values between 1.2 and 2.0. The peak occurs around 1.5, indicating that most molecules in the dataset have a moderate level of branching and complexity. The distribution tapers off significantly after 2.0, suggesting fewer molecules with higher branching complexity. The Schultz MTI distribution is also right-skewed, with a peak around 5000. Most molecules have values between 3000 and 10,000, indicating a moderate to high level of molecular branching and connectivity. The distribution extends up to 20,000, but very few molecules reach these higher values, indicating that extremely complex branching is less common in this dataset. The Szeged index distribution is right-skewed, peaking around 2000. Most molecules have values between 1000 and 4000, indicating that the pairwise distances between vertices in the molecular graph are generally moderate. The distribution extends to about 8000, but higher values are less frequent, suggesting fewer molecules with extensive distances between vertex pairs. The Xu index distribution appears relatively symmetric, with a peak around 10. Most molecules have values between 7 and 14, indicating a moderate level of connectivity and structural influence from vertex degrees. The distribution suggests a balanced presence of molecules with varying levels of vertex connectivity within this range. The molecular complexity distribution, based on Böttcher’s definition is roughly symmetric, with a peak around 300. Most molecules have values between 200 and 400, indicating a moderate level of structural complexity. The distribution shows that the dataset has a balanced representation of molecules with different complexity levels, with fewer molecules at the extreme low and high ends. The entropy distribution is highly concentrated around a single value, close to 2.95. This indicates that almost all molecules in the dataset have very similar entropy values, suggesting a high degree of uniformity in this particular descriptor across the dataset. The narrow range of entropy values implies limited variability in this aspect of the molecular structures.

Most descriptors show a right-skewed distribution, indicating that the majority of molecules have moderate values for these indices, with fewer molecules exhibiting extreme structural features. The uniform distribution of entropy suggests that this particular descriptor might not vary significantly across the dataset. These observations will guide the application of nonparametric tests to determine the impact of these descriptors on clustering results.

### 3.2. Clustering Results

In this section, we present the clustering results obtained from applying four different deep learning methodologies: Deep Belief Network (DBN), Convolutional Autoencoder (CAE), Variational Autoencoder (VAE), and Adversarial Autoencoder (AAE). For each of these deep learning architectures, we employed k-means clustering with cluster sizes of 16, 32, and 64. The results showcase the effectiveness of each method in grouping the molecules based on their spectral geometric properties. By comparing these results, we aim to identify the most suitable deep learning approach and cluster size for capturing the inherent structural patterns in the molecular dataset. Detailed interpretations of the clustering outcomes will provide insights into the performance and robustness of each methodology in the context of molecular clustering.

Table 1 highlights the clustering performance for DBNs with different partitions.

The Calinski–Harabasz index is used to evaluate the quality of clustering by measuring the ratio of between-cluster dispersion to within-cluster dispersion. Higher values indicate better-defined clusters. For the DBN + k-means results, we observe that with K=16 clusters, the index is 1532.07, indicating a moderate level of cluster separation. As the number of clusters increases to K=32, the index rises to 2175.96, suggesting an improvement in the separation and definition of the clusters. With K=64 clusters, the index significantly increases to 14,471.6, indicating that the clusters are exceptionally well-defined and separated. This trend demonstrates that increasing the number of clusters leads to better-defined clusters as measured by the Calinski–Harabasz index.

The Davies–Bouldin index assesses the average similarity ratio of each cluster with its most similar cluster, with lower values indicating better clustering. For K=16 clusters, the index is 0.44316, suggesting that the clusters are relatively compact and well separated. When the number of clusters is increased to K=32, the index decreases to 0.35608, indicating improved compactness and separation of clusters. At K=64, the index further decreases to 0.19196, showing that the clusters are even more compact and well separated. This decreasing trend highlights the effectiveness of using a higher number of clusters for achieving better clustering results.

The Dunn index measures the ratio of the minimum inter-cluster distance to the maximum intra-cluster distance, with higher values indicating better clustering. For K=16 clusters, the index is 0.04986, reflecting a relatively low separation between clusters. As the number of clusters increases to K=32, the index improves to 0.112182, indicating better cluster separation. With K=64, the index significantly improves to 0.4838, suggesting a high degree of separation between clusters. This substantial improvement with more clusters indicates that higher cluster counts contribute to better-defined and more separated clusters.

R-Squared measures the proportion of variance explained by the clustering, with higher values indicating better clustering. For K=16 clusters, the R-Squared value is 0.94382, suggesting that a large portion of the variance in the data is explained by the clustering. When the number of clusters is increased to K=32, the R-Squared value rises to 0.9737, showing an improved explanation of the data variance. At K=64, the R-Squared value further increases to 0.99523, indicating that nearly all the variance in the data is captured by the clustering. This trend demonstrates that a higher number of clusters leads to a more comprehensive explanation of the data variance.

The Silhouette Score evaluates how similar a point is to its own cluster compared to other clusters, with values ranging from −1 to 1 and higher values indicating better clustering. For K=16 clusters, the score is 0.52044, suggesting good clustering quality. As the number of clusters increases to K=32, the score slightly decreases to 0.4989, indicating a slight decline in clustering quality. At K=64, the score further decreases to 0.43674, suggesting a more noticeable decline in clustering quality with a higher number of clusters. This decreasing trend indicates that while more clusters may provide better-defined clusters, the overall cohesion and separation measured by the Silhouette Score may slightly decline.

The Standard Deviation measures the spread of points within each cluster, with lower values indicating more compact clusters. For K=16 clusters, the standard deviation is 1.8814, suggesting a relatively high spread within clusters. As the number of clusters increases to K=32, the standard deviation decreases to 1.0811, indicating more compact clusters. At K=64, the standard deviation significantly decreases to 0.28871, showing highly compact clusters. This trend demonstrates that increasing the number of clusters leads to more compact clustering.

Overall, the clustering results for DBN + k-means indicate that increasing the number of clusters generally improves cluster separation and definition as measured by the Calinski–Harabasz, Davies–Bouldin, Dunn, and R-Squared indices. However, the Silhouette Score suggests a slight decline in clustering quality with a higher number of clusters. The standard deviation shows that clusters become more compact with an increasing number of clusters.

The clustering performance for CAE with different partitions is highlighted in Table 2.

The CAE + k-means findings show that with K=16 clusters, the index is 1465.42, which suggests a moderate level of cluster separation. When the number of clusters is raised to K=32, the index climbs to 1984.89, indicating enhanced cluster delineation. When using 64 clusters, the index experiences a substantial increase to 11,659.3, suggesting the presence of highly distinct and well-defined clusters. The observed pattern indicates that a higher number of clusters generally improves the distinction across clusters, as quantified by the Calinski–Harabasz index.

With 16 clusters, the index is 0.43703, indicating that the clusters are relatively compact and well separated. When the number of clusters is raised to K=32, the index lowers slightly to 0.3888, suggesting an enhancement in the compactness and separation of the clusters. When K=64, the index lowers to 0.253, indicating that the clusters become more compact and distinct with 64 clusters. The declining pattern emphasizes the efficacy of employing a larger number of clusters to obtain superior clustering outcomes.

With 16 clusters, the index is 0.08787, indicating a rather low level of separation between the clusters. As the number of clusters increases to K=32, the index lowers to 0.03924, suggesting a decrease in the separation between clusters. When K=64, the index is 0.0264, indicating that there is still room for more reduction in cluster separation. This trend suggests that as the number of clusters rises, their compactness increases while their distance from each other decreases.

With 16 clusters, the R-Squared value is 0.9446, indicating that a significant amount of the variability in the data can be accounted for by the grouping. By increasing the number of clusters to K=32, the R-Squared value increases to 0.9728, indicating a higher level of explanation for the variance in the data. When K=64, the R-Squared value increases even more to 0.995, which suggests that almost all of the variability in the data is accounted for by the clustering. This trend illustrates that an increased number of clusters results in a more thorough elucidation of the variance in the data.

The clustering quality is considered good with a score of 0.5893 for 16 clusters. When the number of clusters is increased to K=32, the score lowers slightly to 0.5549, suggesting a minor decrease in the quality of clustering. When K=64, the score lowers even further to 0.3656, indicating a significant decrease in the quality of clustering when there are more clusters. This declining trend suggests that increasing the number of clusters may result in more distinct clusters, but it may also lead to a decrease in the overall cohesiveness and separation as measured by the Silhouette Score.

With K=16 clusters, the standard deviation is 2.1218, indicating a significant dispersion among the clusters. As the number of clusters increases to K=32, the standard deviation drops to 1.2206, suggesting a higher level of cluster compactness. When K=64, the standard deviation reduces significantly to 0.3887, indicating the presence of highly compact clusters. This pattern illustrates that augmenting the quantity of clusters results in clustering that is more condensed.

To summarize, the clustering outcomes for CAE + k-means demonstrate that augmenting the number of clusters typically enhances the distinction and precision of the clusters, as assessed by the Calinski–Harabasz and Davies–Bouldin indices. Nevertheless, the Dunn index indicates that as the number of clusters increases, there is a reduction in the spacing between the clusters. The R-Squared values exhibit a positive trend, suggesting a more effective explanation of the variability in the data as the number of clusters increases. The Silhouette Score demonstrates a negative correlation between clustering quality and the number of clusters, whereas the standard deviation suggests an increase in cluster compactness.

Table 3 highlights the clustering performance for VAE with different partitions.

The VAE + k-means results, with K=16 clusters, yield an index of 1432.01, indicating a considerable degree of cluster separation. When the number of clusters is increased to K=32, the index shows a considerable increase to 3807.64, suggesting a notable enhancement in the definition of the clusters. When the number of clusters is increased to K=64, the index rises to 5630.58, indicating that the clusters become more distinct and separated with a larger number of clusters. The observed pattern indicates that augmenting the number of clusters improves the distinction between clusters, as quantified by the Calinski–Harabasz index.

With 16 clusters, the index is 0.446, suggesting that the clusters are relatively compact and well separated. As the number of clusters increases to K=32, the index lowers to 0.3958, indicating enhanced cluster separation and compactness. When K=64, the index lowers to 0.2166, indicating that the clusters become more compact and distinct from each other. The declining pattern emphasizes the efficacy of employing a larger number of clusters to attain superior clustering outcomes.

With 16 clusters, the index is 0.0219, indicating a rather low level of separation between the clusters. As the number of clusters increases to K=32, the index improves to 0.0614, suggesting enhanced cluster separation. When K=64, the index experiences a minor fall to 0.0271, indicating a decrease in the spacing between clusters. This pattern suggests that although having more clusters may result in improved early differentiation, it becomes increasingly difficult to sustain this differentiation as the number of clusters continues to grow.

With 16 clusters, the R-Squared value is 0.9444, indicating that a significant proportion of the variability in the data can be accounted for by the grouping. By increasing the number of clusters to K=32, the R-Squared value increases to 0.9785, indicating a higher level of explanation for the variance in the data. When K=64, the R-Squared value increases even more to 0.9932, suggesting that almost all of the variability in the data is accounted for by the clustering. This trend illustrates that an increased number of clusters results in a more thorough elucidation of the variance in the data.

The clustering quality is considered good, with a score of 0.576 for K=16 clusters. When the number of clusters is increased to K=32, the score lowers slightly to 0.5267, suggesting a minor decrease in the quality of clustering. When K=64, the score reduces to 0.3951, indicating a significant decrease in the quality of clustering as the number of clusters increases. This declining trend suggests that increasing the number of clusters may result in more distinct clusters, but it may also lead to a decrease in the overall cohesiveness and separation as measured by the Silhouette Score.

With K=16 clusters, the standard deviation is 1.5746, indicating a significant dispersion among the clusters. As the number of clusters increases to K=32, the standard deviation reduces to 0.663, suggesting a higher level of cluster compactness. When K=64, the standard deviation reduces significantly to 0.371, indicating the presence of highly compact clusters. This pattern illustrates that augmenting the quantity of clusters results in clustering that is more condensed.

In general, the clustering results for VAE + k-means suggest that increasing the number of clusters enhances the separation and definition of the clusters, as evaluated by the Calinski–Harabasz and Davies–Bouldin indices. The Dunn index demonstrates an initial enhancement in the distinction between clusters but experiences a little deterioration as the number of clusters becomes excessively high. The R-Squared values exhibit a positive trend, suggesting a more effective explanation of the variability in the data as the number of clusters increases. The Silhouette Score demonstrates a negative correlation between clustering quality and the number of clusters, whereas the standard deviation suggests an increase in cluster compactness.

The clustering performance for AAE with different partitions is highlighted in Table 4.

The AAE + k-means results show a moderate level of cluster separation with an index of 1090.47 for K=16 clusters. The index climbs to 2744.92 when the number of clusters is increased to K=32, indicating a significant improvement in cluster definition. Clusters with K=64 show exceptionally well-defined and distinct features, as the index surges to 52,322.5. This pattern suggests that the Calinski–Harabasz index measures cluster separation better as the number of clusters increases.

The index for clusters with K = 16 is 0.3801, suggesting that the clusters are moderately compact and well separated. A drop in the index to 0.2897 at K=32 indicates better cluster separation and compactness as the number of clusters is raised. The clusters become increasingly more compact and well separated when the index drops to 0.1157 at K=64. It is clear from this declining trend that increasing the number of clusters yields better clustering outcomes.

As a result, the index for clusters with K = 16 is 0.1761, which indicates a modest level of separation between them. With a higher number of clusters (K = 32), the index improves to 0.2129, suggesting that the clusters are better separated. A high level of cluster separation is shown by the index’s considerable increase to 0.5673 at K=64. This pattern shows that more clusters result in more distinct and well-defined clusters.

The clustering explains a significant amount of the data variation, as indicated by the R-Squared value of 0.9343 for K=16 clusters. An enhanced explanation of the data variance is demonstrated by an R-Squared value of 0.9774 when the number of clusters is raised to K=32. The R-Squared value rises to 0.9978 at K=64, suggesting that clustering accounts for almost all of the data variance. As we can see from this pattern, adding more clusters helps to explain the data variance more thoroughly.

The score of 0.5026 indicates acceptable clustering quality for clusters with K = 16. With an increase in the number of clusters to 32, the score drops to 0.5033, suggesting that the clustering quality remains consistent. The score drops even lower to 0.4793 for K=64, indicating that clustering quality slightly declines as the number of clusters increases. Although a larger number of clusters may result in more clearly defined clusters, this trend suggests that the Silhouette Score’s overall measure of group cohesiveness and separation may slightly decrease.

The standard deviation of 2.0263 for clusters with K = 16 indicates a reasonably high spread within clusters. With an increase in the number of clusters to 32, the standard deviation drops to 0.9135, suggesting that the clusters are becoming more compact. Highly compact clusters are indicated by a considerable decrease in the standard deviation to 0.1435 at K=64. Increasing the number of clusters causes clustering to become more compact, as shown by this pattern.

In general, the Calinski–Harabasz, Davies–Bouldin, and Dunn indices show that increasing the number of clusters generally enhances cluster separation and definition, according to the clustering results for AAE + k-means. An upward trend in the R-Squared values suggests that more clusters provide a more satisfactory explanation of the data variation. Standard deviation demonstrates that clusters become denser as cluster size increases, while Silhouette Score shows stable clustering quality with a small drop at maximum cluster size.

### 3.3. Nonparametric Tests

To comprehensively evaluate the impact of topological descriptors on the clustering results, we will employ nonparametric statistical tests. These tests are particularly suitable given the nature of our data, which may not adhere to normal distribution assumptions. By applying nonparametric tests, we aim to rigorously assess whether the topological indices significantly influence the formation and quality of clusters across different clustering methodologies. Specifically, we will analyze the effects of topological descriptors on the clusters generated by Deep Belief Network (DBN), Convolutional Autoencoder (CAE), Variational Autoencoder (VAE), and Adversarial Autoencoder (AAE) combined with k-means clustering. The test findings yield vital insights into the structural factors that determine molecular clustering. Additionally, they will emphasize the strength and dependability of each clustering approach in accurately representing the underlying topological features of the molecules.

We present the nonparametric test results in Table 5 for clusters determined by the DBN.

Table 5 presents the nonparametric test results for the effect of topological descriptors on clusters formed by the Deep Belief Network (DBN) combined with k-means clustering. The tests performed include the Kruskal–Wallis test, the Conover post hoc test, and the Log-Rank test. These tests evaluate the significance of the differences in topological indices across clusters for different numbers of clusters (K = 16, 32, 64).

For K=16 clusters, the Kruskal–Wallis test shows significant results for Balaban J, indicating that there are differences in this descriptor across clusters. The Conover post hoc test also shows significance for Balaban J. The Log-Rank test supports these findings with a *p*-value. Other descriptors such as Schultz MTI, Szeged, Xu, Complexity, and Entropy of AC do not show significant differences across clusters, as indicated by higher *p*-values.

When the number of clusters increases to K=32, the Kruskal–Wallis test indicates significant differences for Schultz MTI, while other descriptors do not show significant results. The Conover test reveals some significance for Schultz MTI and Szeged, indicating that these descriptors have significant differences across clusters. The Log-Rank test supports the significance for Schultz MTI, confirming that this descriptor varies significantly across clusters.

For K=64 clusters, the Kruskal–Wallis test does not show significant results for any of the topological descriptors, as all *p*-values are well above 0.05. This indicates that there are no statistically significant differences in the distribution of these descriptors across the clusters. The Conover and Log-Rank tests similarly do not show significant results for most descriptors, confirming the findings of the Kruskal–Wallis test. Overall, for K=64 clusters, the nonparametric tests indicate that there are no significant differences in the topological descriptors across clusters. This suggests that, with a high number of clusters, the impact of topological descriptors on clustering is not statistically significant according to these tests. This result highlights the complexity and potential limitations of using very high numbers of clusters to discern the impact of topological indices on clustering results.

Comparing the results across different numbers of clusters, it is evident that for K=16 clusters, only Balaban J shows significant differences across clusters, indicating its impact on clustering. For K=32 clusters, multiple descriptors such as Schultz MTI, Szeged, and Complexity show significant differences, suggesting a stronger impact of these descriptors on clustering as the number of clusters increases. However, for K=64 clusters, none of the descriptors show significant differences across clusters, indicating that the high number of clusters may dilute the impact of individual topological descriptors on the clustering results.

The nonparametric test findings for clusters defined by CEA are displayed in Table 6.

For K=16 clusters, the Kruskal–Wallis test shows no significant results for any of the topological descriptors, as indicated by high *p*-values (e.g., Balaban J: p=0.63078, Schultz MTI: p=0.066489, Szeged: p=0.457867). The Conover and Log-Rank tests also confirm these findings with high *p*-values, indicating that the topological descriptors do not significantly impact the clustering results for this number of clusters.

When the number of clusters increases to K=32, the Kruskal–Wallis test again shows no significant results for any of the descriptors, with high *p*-values (e.g., Balaban J: p=0.81469, Schultz MTI: p=0.840177, Complexity: p=0.849945). The Conover test reveals lower *p*-values for Schultz MTI, but they are still not significant. The Log-Rank test similarly shows high *p*-values for all descriptors, indicating that the impact of topological descriptors remains limited for K=32 clusters.

For K=64 clusters, the Kruskal–Wallis test does not show significant results for any of the topological descriptors, as all *p*-values are high. The Conover test confirms these findings with high *p*-values for all descriptors, such as Balaban J, Schultz MTI, and Complexity. The Log-Rank test also supports these results with high *p*-values, indicating no significant impact of the topological descriptors on the clustering results when there are 64 clusters.

Comparing the results across different numbers of clusters, it is evident that for K=16 clusters, none of the topological descriptors show significant differences across clusters, indicating that the clustering structure is not strongly influenced by topological features. For K=32 clusters, there are still no significant differences in any of the descriptors. For K=64 clusters, none of the descriptors show significant differences across clusters, suggesting that the high number of clusters does not reveal significant impacts of topological descriptors.

The nonparametric test results for CAE + k-means clustering reveal that topological descriptors such as Balaban J, Schultz MTI, Szeged, Xu, Complexity, and Entropy of AC do not significantly influence the clustering results for K=16, K=32, and K=64 clusters. These findings suggest that the clustering structure is not significantly affected by the topological descriptors used in this analysis, highlighting the complexity and potential limitations of using these descriptors to discern the impact on clustering results.

We present the nonparametric test results in Table 7 for clusters determined by VAE.

Based on the high *p*-values (e.g., Balaban J: p=0.321723, Schultz MTI: p=0.207822, Szeged: p=0.515028), the Kruskal–Wallis test does not yield any significant results for any of the topological descriptors in the K=16 clusters. These findings are also confirmed by the Conover and Log-Rank tests, which yield high *p*-values. This suggests that the topological descriptors do not have a substantial influence on the clustering results for this number of clusters.

The Kruskal–Wallis test yields no significant results for any of the descriptors when the number of clusters reaches K=32, despite the high *p*-values. The *p*-values for Complexity are lower in the Conover test (p=0.106529), but they are still not statistically significant. The Log-Rank test also yields high *p*-values for all descriptors, suggesting that the influence of topological descriptors is restricted to K=32 clusters.

The Kruskal–Wallis test does not yield significant results for any of the topological descriptors in the K=64 clusters, as all *p*-values are high (e.g., Balaban J: p=0.629391, Schultz MTI: p=0.726976, Szeged: p=0.267479, Xu: p=0.28601). These findings are corroborated by the Conover test, which yields high *p*-values for all descriptors, including Balaban J (p=0.067499), Schultz MTI (p=0.630139), and Complexity (p=0.07102). The Log-Rank test also supports these results with high *p*-values, suggesting that the topological descriptors do not have a significant impact on the clustering results when there are 64 clusters.

It is clear that the clustering structure is not significantly influenced by topological features when comparing the results across different numbers of clusters, as none of the topological descriptors exhibit significant differences across clusters for K=16. There are no significant differences in any of the descriptors for K=32 clusters. None of the descriptors exhibit significant disparities across clusters for K=64 clusters, indicating that the high number of clusters does not reveal significant impacts of topological descriptors.

The clustering results for the K=16, K=32, and K=64 clusters are not significantly influenced by topological descriptors such as Balaban J, Schultz MTI, Szeged, Xu, Complexity, and Entropy of AC, as confirmed by the nonparametric test results for VAE + k-means clustering. These results indicate that the topological descriptors employed in this analysis do not have a substantial impact on the clustering structure, underscoring the potential limitations and complexity of utilizing these descriptors to determine their impact on clustering results.

The nonparametric test findings for clusters defined by AAE are displayed in Table 8.

For K=16 clusters, the Kruskal–Wallis test shows no significant results for any of the topological descriptors, as indicated by high *p*-values (e.g., Balaban J: p=0.757166, Schultz MTI: p=0.710266, Szeged: p=0.0654255). The Conover and Log-Rank tests also confirm these findings with high *p*-values, indicating that the topological descriptors do not significantly impact the clustering results for this number of clusters.

When the number of clusters increases to K=32, the Kruskal–Wallis test shows no significant results for any of the descriptors, with high *p*-values (e.g., Balaban J: p=0.291994, Schultz MTI: p=0.371202, Szeged: p=0.863761). The Conover test reveals lower *p*-values for Balaban J (p=0.195614), but they are still not significant. The Log-Rank test similarly shows high *p*-values for all descriptors, indicating that the impact of topological descriptors remains limited for K=32 clusters.

For K=64 clusters, the Kruskal–Wallis test does not show significant results for any of the topological descriptors, as all *p*-values are high (e.g., Balaban J: p=0.503463, Schultz MTI: p=0.127896, Szeged: p=0.540773, Xu: p=0.161865). The Conover test, however, shows significant results for Balaban J (p=0.0212421), indicating some impact of this descriptor on the clustering results. The Log-Rank test does not support significant results for most descriptors, confirming the high *p*-values for Balaban J (p=0.449241), Schultz MTI (p=0.142711), and Szeged (p=0.529786).

Comparing the results across different numbers of clusters, it is evident that for K=16 clusters, none of the topological descriptors show significant differences across clusters, indicating that the clustering structure is not strongly influenced by topological features. For K=32 clusters, there are still no significant differences in any of the descriptors. For K=64 clusters, only Balaban J shows some significance according to the Conover test (p=0.0212421), but other tests do not confirm this significance. This suggests that the high number of clusters may reveal some impact of individual topological descriptors on the clustering results, though the overall impact remains limited.

The nonparametric test results for AAE + k-means clustering reveal that topological descriptors such as Balaban J, Schultz MTI, Szeged, Xu, Complexity, and Entropy of AC do not significantly influence the clustering results for K=16 and K=32 clusters. For K=64 clusters, the Conover test indicates some significance for Balaban J, but other tests do not confirm this. These findings suggest that the clustering structure is generally not significantly affected by the topological descriptors used in this analysis.

## 4. Conclusions

This study presents an advanced spectral geometric methodology for clustering a large-scale molecular library. The process involves converting molecular structures into triangulated meshes, applying the Laplace–Beltrami operator to capture intrinsic geometric features, and employing weighted sampling based on discrete mean curvature to prioritize significant structural characteristics. This approach allows for a detailed examination of molecular surfaces, aiding in the classification and clustering of molecules.

Our evaluation using various clustering metrics, such as the Calinski–Harabasz index and Davies–Bouldin index, reveals that increasing the number of clusters generally improves separation and definition, although it can lead to a decline in cohesion as indicated by the Silhouette Score. The standard deviation of cluster sizes suggests that as clusters become more uniform, there is a potential trade-off in clustering quality.

Among the methodologies tested, the combination of Deep Belief Networks (DBNs) with k-means clustering emerged as the most effective, particularly at lower cluster counts (K = 16, K = 32). This method consistently produced well-defined and distinct clusters, with high sensitivity to structural variations in the molecular data. Nonparametric tests further validated the method’s effectiveness, demonstrating significant differences in topological descriptors such as the Balaban J and Schultz MTI indices.

Our methodology addresses several limitations associated with traditional clustering techniques like k-means, hierarchical clustering, and DBSCAN, particularly their reliance on predefined distance metrics and their inability to capture the complex relationships within molecular structures. By leveraging spectral geometry, we move beyond simple Euclidean distance measures and instead utilize the Laplace–Beltrami operator to extract eigenvalues and eigenvectors that represent the intrinsic geometric features of molecular surfaces. Additionally, the integration of curvature-based weighted sampling ensures that the most structurally significant regions of the molecule are prioritized during the clustering process. Finally, the incorporation of Deep Belief Networks (DBNs) allows our approach to model nonlinear relationships between atoms and bonds, capturing the nuanced interactions that traditional methods often miss. These innovations collectively enable our method to provide more accurate and meaningful clustering results, particularly when dealing with the complex and diverse nature of molecular datasets.

While pronounced curvature is a practical indicator of structural complexity in many cases, we acknowledge that it may not capture all chemically significant features, such as the aromatic pi electron cloud in benzene. Despite this, our curvature-based sampling remains computationally efficient and effective for large-scale studies, with future work aimed at integrating additional electronic descriptors for a more holistic analysis.

In conclusion, the DBN + k-means combination offers robust and reliable clustering performance, making it a powerful tool for analyzing complex molecular structures. Future research should explore the integration of more diverse topological descriptors, advanced feature selection techniques, and alternative clustering methods to further enhance the methodology’s robustness and scalability.

## Figures and Tables

**Table 1:** Clustering measurements for DBN + k-means.

	K Partitions		
	**16**	**32**	**64**
**Calinski–Harabasz**	1532.07	2175.96	14,471.6
**Davies–Bouldin**	0.44316	0.35608	0.19196
**Dunn**	0.04986	0.112182	0.4838
**R Squared**	0.94382	0.9737	0.99523
**Silhouette**	0.52044	0.4989	0.43674
**Std. Deviation**	1.8814	1.0811	0.28871

**Table 2:** Clustering measurements for CAE + k-means.

	K Partitions		
	**16**	**32**	**64**
**Calinski–Harabasz**	1465.42	1984.89	11,659.3
**Davies–Bouldin**	0.43703	0.3888	0.253
**Dunn**	0.08787	0.03924	0.0264
**R Squared**	0.9446	0.9728	0.995
**Silhouette**	0.5893	0.5549	0.3656
**Std. Deviation**	2.1218	1.2206	0.3887

**Table 3:** Clustering measurements for VAE + k-means.

	K Partitions		
	**16**	**32**	**64**
**Calinski–Harabasz**	1432.01	3807.64	5630.58
**Davies–Bouldin**	0.446	0.3958	0.2166
**Dunn**	0.0219	0.0614	0.0271
**R Squared**	0.9444	0.9785	0.9932
**Silhouette**	0.576	0.5267	0.3951
**Std. Deviation**	1.5746	0.663	0.371

**Table 4:** Clustering measurements for AAE + k-means.

	K Partitions		
	**16**	**32**	**64**
**Calinski–Harabasz**	1090.47	2744.92	52,322.5
**Davies–Bouldin**	0.3801	0.2897	0.1157
**Dunn**	0.1761	0.2129	0.5673
**R-Squared**	0.9343	0.9774	0.9978
**Silhouette**	0.5026	0.5033	0.4793
**Std. Deviation**	2.0263	0.9135	0.1435

**Table 5:** Nonparametric test results for DBN + k-means.

			Balaban J	Schultz MTI	Szeged	Xu	Complexity	Ent. of AC
**K = 16**	**Kruskal–Wallis**	**Statistic**	24.7155	13.5141	5.73053	13.2394	15.1062	5.49284
		***p*** **-value**	0.053726	0.562882	0.983985	0.584049	0.443926	0.987115
	**Conover**	**Statistic**	24.371	11.8065	14.331	15.285	12.7754	5.48873
		***p*** **-value**	0.0590545	0.693612	0.500589	0.431086	0.619642	0.987117
	**Log-Rank**	**Statistic**	24.9406	13.6281	5.68998	13.1921	15.3498	5.49194
		***p*** **-value**	0.0507473	0.553897	0.984498	0.587466	0.426525	0.987078
**K = 32**	**Kruskal–Wallis**	**Statistic**	19.4682	46.863	26.8608	1.40448	29.2374	24.3715
		***p*** **-value**	0.946687	0.0335663	0.679407	0.0671132	0.557048	0.795374
	**Conover**	**Statistic**	43.4298	38.908	47.6954	35.608	42.7937	24.3657
		***p*** **-value**	0.0682896	0.15556	0.0281405	0.260339	0.0772623	0.795343
	**Log-Rank**	**Statistic**	20.1159	46.7215	26.9914	42.068	27.9231	24.3675
		***p*** **-value**	0.933287	0.0347522	0.672608	0.0886842	0.625132	0.795267
**K = 64**	**Kruskal–Wallis**	**Statistic**	74.9952	60.1045	80.2071	57.6369	52.3111	67.7942
		***p*** **-value**	0.143001	0.580341	0.0705459	0.667426	0.829673	0.317022
	**Conover**	**Statistic**	74.2187	83.6774	91.9865	53.9723	72.3119	67.8002
		***p*** **-value**	0.157639	0.0418117	0.0100435	0.784084	0.197486	0.316928
	**Log-Rank**	**Statistic**	75.2593	60.4183	80.3978	57.3542	53.3881	67.8008
		***p*** **-value**	0.138535	0.568899	0.0688042	0.676862	0.800655	0.316912

**Table 6:** Nonparametric test results for CEA + k-means.

			Balaban J	Schultz MTI	Szeged	Xu	Complexity	Ent. of AC
**K = 16**	**Kruskal–Wallis**	**Statistic**	12.6344	23.9083	14.9127	11.577	11.0135	11.7364
		***p*** **-value**	0.63078	0.066489	0.457867	0.711014	0.751932	0.699154
	**Conover**	**Statistic**	19.4972	16.4604	14.7809	6.81564	5.54564	11.7394
		***p*** **-value**	0.19208	0.352128	0.46731	0.962565	0.986412	0.698641
	**Log-Rank**	**Statistic**	13.0265	24.1589	14.9264	11.4943	11.1073	11.737
		***p*** **-value**	0.600251	0.062444	0.456733	0.716834	0.744948	0.698819
**K = 32**	**Kruskal–Wallis**	**Statistic**	23.9047	23.2503	30.3422	29.6951	22.9849	20.1919
		***p*** **-value**	0.81469	0.84017	0.49981	0.533237	0.849945	0.931781
	**Conover**	**Statistic**	21.9212	30.8815	34.6184	34.9957	27.4156	20.1951
		***p*** **-value**	0.885472	0.472195	0.29917	0.283973	0.651146	0.931529
	**Log-Rank**	**Statistic**	23.5147	23.2601	30.3271	29.6777	23.5961	20.1928
		***p*** **-value**	0.829837	0.839536	0.500454	0.533984	0.826675	0.93158
**K = 64**	**Kruskal–Wallis**	**Statistic**	73.2679	69.1039	65.7806	62.5305	67.526	75.9931
		***p*** **-value**	0.176516	0.278772	0.380774	0.493088	0.325182	0.125932
	**Conover**	**Statistic**	75.5143	103.449	61.072	51.2705	79.5678	75.983
		***p*** **-value**	0.13413	0.000998	0.545362	0.854856	0.077565	0.126309
	**Log-Rank**	**Statistic**	72.7551	69.6429	66.1372	62.8398	72.7092	75.9879
		***p*** **-value**	0.18766	0.263981	0.369126	0.481988	0.188659	0.126229

**Table 7:** Nonparametric test results for VAE + k-means.

			Balaban J	Schultz MTI	Szeged	Xu	Complexity	Ent. of AC
**K = 16**	**Kruskal–Wallis**	**Statistic**	16.9523	19.1306	14.1401	8.79463	13.7757	14.2528
		***p*** **-value**	0.321723	0.207822	0.515028	0.888121	0.542711	0.506542
	**Conover**	**Statistic**	16.3565	14.6992	16.3388	22.8889	8.32586	14.2576
		***p*** **-value**	0.358762	0.473292	0.359897	0.086526	0.910055	0.50609
	**Log-Rank**	**Statistic**	16.8656	19.1617	14.1999	8.77269	15.1248	14.2539
		***p*** **-value**	0.32696	0.206501	0.510428	0.8891	0.442467	0.506368
**K = 32**	**Kruskal–Wallis**	**Statistic**	28.0932	28.6905	44.0416	30.5014	47.3591	25.3266
		***p*** **-value**	0.616462	0.585448	0.0604174	0.491588	0.030214	0.753058
	**Conover**	**Statistic**	41.0756	21.7749	41.0772	28.114	24.3776	25.3283
		***p*** **-value**	0.106529	0.889957	0.106499	0.615274	0.79484	0.752843
	**Log-Rank**	**Statistic**	27.3829	29.0108	43.9137	30.5661	46.5767	25.3249
		***p*** **-value**	0.65281	0.568671	0.0620703	0.488224	0.035845	0.753
**K = 64**	**Kruskal–Wallis**	**Statistic**	58.7257	55.8446	69.5124	68.8509	55.9449	62.4533
		***p*** **-value**	0.629391	0.726976	0.267479	0.28601	0.723748	0.4958
	**Conover**	**Statistic**	80.5288	60.3193	82.2275	54.5488	85.9249	62.4451
		***p*** **-value**	0.0674999	0.572459	0.0523603	0.767102	0.029077	0.49605
	**Log-Rank**	**Statistic**	59.7582	56.0746	69.4219	68.5358	58.6044	62.448
		***p*** **-value**	0.592591	0.719429	0.270038	0.29514	0.633562	0.495947

**Table 8:** Nonparametric test results for AAE + k-means.

			Balaban J	Schultz MTI	Szeged	Xu	Complexity	Ent. of AC
**K = 16**	**Kruskal–Wallis**	**Statistic**	10.9395	11.5871	23.97	12.7769	8.53363	19.5261
		***p*** **-value**	0.757166	0.710266	0.0654255	0.619789	0.900825	0.19075
	**Conover**	**Statistic**	18.086	18.2739	24.9079	18.6645	24.4969	19.5261
		***p*** **-value**	0.258172	0.24854	0.0512	0.229339	0.0571214	0.190877
	**Log-Rank**	**Statistic**	10.8737	11.4223	24.3165	12.7301	8.68641	19.5261
		***p*** **-value**	0.761496	0.722122	0.0599	0.623136	0.893335	0.190877
**K = 32**	**Kruskal–Wallis**	**Statistic**	34.7931	32.9638	22.5931	34.4058	22.6418	42.4006
		***p*** **-value**	0.291994	0.371202	0.863761	0.30788	0.862085	0.08309
	**Conover**	**Statistic**	37.5	22.9603	33.4921	43.3172	24.1862	42.4006
		***p*** **-value**	0.195614	0.850573	0.347294	0.06981	0.802872	0.083286
	**Log-Rank**	**Statistic**	34.7966	33.3147	22.5072	34.8518	23.1819	42.4006
		***p*** **-value**	0.291934	0.35523	0.866442	0.289713	0.842455	42.4006
**K = 64**	**Kruskal–Wallis**	**Statistic**	62.2405	75.8732	61.2031	73.9908	66.5786	74.8472
		***p*** **-value**	0.503463	0.127896	0.540773	0.161865	0.354849	0.145672
	**Conover**	**Statistic**	87.7873	76.3364	74.3757	60.522	62.9508	74.8531
		***p*** **-value**	0.0212421	0.120648	0.15464	0.56517	0.478048	0.145774
	**Log-Rank**	**Statistic**	63.7687	75.0231	61.5046	73.1647	68.5528	74.8449
		***p*** **-value**	0.449241	0.142711	0.529786	0.1789	0.294645	0.145925

## Data Availability

The original contributions presented in this study are included in the article; further inquiries can be directed to the corresponding author.
